# TOP2A correlates with poor prognosis and affects radioresistance of medulloblastoma

**DOI:** 10.3389/fonc.2022.918959

**Published:** 2022-07-15

**Authors:** Yufeng Zhang, Haiyan Yang, Liwen Wang, Huandi Zhou, Ge Zhang, Zhiqing Xiao, Xiaoying Xue

**Affiliations:** ^1^ Department of Radiotherapy, The Second Hospital of Hebei Medical University, Shijiazhuang, China; ^2^ Department of Pathology, The Second Hospital of Hebei Medical University, Shijiazhuang, China

**Keywords:** medulloblastoma, TOP2A, prognosis, radioresistance, Wnt/β-catenin pathway

## Abstract

Radiotherapy remains the standard treatment for medulloblastoma (MB), and the radioresistance contributes to tumor recurrence and poor clinical outcomes. Nuclear DNA topoisomerase II-alpha (TOP2A) is a key catalytic enzyme that initiates DNA replication, and studies have shown that TOP2A is closely related to the therapeutic effects of radiation. In this study, we found that TOP2A was significantly upregulated in MB, and high expression of TOP2A related to poor prognosis of MB patients. Knockdown of TOP2A inhibited MB cell proliferation, migration, and invasion, whereas overexpression of TOP2A enhanced the proliferative and invasive ability of MB cells. Moreover, si-TOP2A transfection in combination with irradiation (IR) significantly reduced the tumorigenicity of MB cells, compared with those transfected with si-TOP2A alone. Cell survival curve analysis revealed that the survival fraction of MB cells was significantly reduced upon TOP2A downregulation and that si-TOP2A-transfected cells had decreased D_0_, Dq, and SF_2_ values, indicating that TOP2A knockdown suppresses the resistance to radiotherapy in MB cells. In addition, western blot analysis demonstrated that the activity of Wnt/β-catenin signaling pathway was inhibited after TOP2A downregulation alone or in combination with IR treatment, whereas overexpression of TOP2A exhibited the opposite effects. Gene set enrichment analysis also revealed that Wnt/β-catenin signaling pathway is enriched in TOP2A high-expression phenotypes. Collectively, these data indicate that high expression of TOP2A leads to poor prognosis of MB, and downregulation of TOP2A inhibits the malignant behaviour as well as the radioresistance of MB cells. The Wnt/β-catenin signaling pathway may be involved in the molecular mechanisms of TOP2A mediated reduced tumorigenicity and radioresistance of MB cells.

## Introduction

Medulloblastoma (MB) is the most common embryonal tumor of the central nervous system, accounting for approximately 20% of all pediatric brain tumors (1). According to molecular characteristics, MB is genetically classified as wingless (WNT)-activated, sonic hedgehog (SHH)-activated TP53 wild-type, SHH-activated TP53 mutant, and non-WNT/non-SHH, which are closely related to the therapy and prognosis of MB (2). The primary treatment for MB is surgery combined with radiotherapy and chemotherapy. The posterior fossa is the most common site of MB, and completely resecting the tumor is often difficult (3). MB could be risk-stratified as standard risk and high risk, depending on clinical factors, such as age, the extent of surgical resection, and the presence of metastasis inside or outside the central nervous system (4). Although advances in surgical operations and radio-and chemotherapy techniques have occurred in recent years, tumor recurrence remains frequent, and approximately one-third of patients die of recurrent tumors (5). Recurrence occurs not only because of the invasiveness and metastasis of the tumor, but also owing to the restrictions on the radiation dose in children’s central nervous system (6). Moreover, the long-term side effects of radiotherapy have prompted researchers to search for methods to reduce the radiation dose.

Nuclear DNA topoisomerase II-alpha (TOP2A), located at 17q21.2, encodes an essential enzyme that regulates chromosome condensation and chromatid separation by altering the topological state of DNA in the process of DNA replication and transcription (7). TOP2A is involved in cell cycle regulation and cell repair. High expression of TOP2A in many types of tumors, such as non-small cell lung cancer, hepatocellular carcinoma, and breast cancer, has been proven to be a dependable proliferation marker and is associated with disease progression and poor prognosis (8–10). Studies have also revealed that TOP2A is upregulated in MB and negatively correlates with the survival time of patients with MB (11, 12). TOP2A has been used as a crucial therapeutic target for tumors, including MBs (12–14).

DNA double-strand breaks are the most lethal form of damage to tumor cells after radiotherapy, and several studies have shown that TOP2A is closely related to the therapeutic effects of radiation. For example, Terry et al. found that the expression level of TOP2A is a factor determining the radiosensitivity of chromatids. TOP2A is indirectly involved in the formation of chromatid breaks from DNA double-stranded breaks (DSBs) induced by radiation, and act as a possible role in the inter-individual variation of chromatid radiosensitivity (15). The study of Kim et al. showed that radiation-induced changes in promoter-CpG islands of TOPO2A can regulate the radioresistance of laryngeal cancer cells; thus, TOPO2A may serve as a useful prognostic indicator for radiotherapy (16). Moreover, in breast cancer, high TOP2A expression is associated with local recurrence after radiotherapy (17). The upregulation of TOP2A also acts as a biomarker for recurrence in prostate cancer patients undergoing radiotherapy (18). In addition, a study of TOP2A single nucleotide polymorphism analysis found that TOP2A rs471692 was not associated with radiotherapy response in lung cancer in Han Chinese individuals (19). Collectively, these studies reveal that TOP2A plays an important role in the process of radiotherapy, and the molecular mechanism downstream of TOP2A during irradiation is rarely reported. Radiotherapy is a necessary postoperative treatment for MB. However, little is known of the functional role of TOP2A in tumorigenesis and radiotherapy of MB.

In this study, we found that TOP2A was significantly upregulated in MB tissues compared with normal brain tissues, and high expression of TOP2A related to poor prognosis of MB patients. Knockdown of TOP2A obviously inhibited MB cell proliferation, migration and invasion, and sensitised tumor cells to radiotherapy. Through colony formation assay, western blot assay and GSEA analysis, we found that downregulation of TOP2A reduced radioresistance of MB cells *via* inhibiting the activity of Wnt/β-catenin signaling pathway. In conclusion, our results suggest that TOP2A plays a critical role in the regulation of irradiation sensitivity and can be used as a potential target to improve radiosensitivity during MB treatment.

## Materials and methods

### Gene expression analysis in public datasets

Six gene expression datasets of MB (GSE50161 (20), GSE39182 (21), GSE74195 (22), GSE35493 (23), GSE85217 (24) and GSE109401 (25)) were downloaded from the Gene Expression Omnibus (GEO, https://www.ncbi.nlm.nih.gov/geo/) database (**Table 1**), and 532 MB samples with complete clinical information in GSE85217 were subjected to prognosis and clinicopathological characteristics analysis (**Table 2**). Expression analysis in paediatric brain tumors was performed using UALCAN (http://ualcan.path.uab.edu/) (26). In addition, the Cancer Cell Line Encyclopedia (CCLE) database (https://portals.broadinstitute.org/ccle/home) (27) was used to assess gene expression in various tumor cell lines.

**Table 1:** Details of selected GEO datasets in this study.

Datasets	Platform	Number of samples			Reference
		Tumor	Normal	Total	
GSE50161	HG-U133_Plus_2	22	13	35	Griesinger et al., 2013 (20)
GSE39182	Agilent-014850	20	5	25	Valdora et al., 2013 (21)
GSE74195	HG-U133_Plus_2	27	5	32	de Bont et al., 2008 (22)
GSE35493	HG-U133_Plus_2	21	9	30	Birks et al., 2013 (23)
GSE85217	HuGene-1_1-st	432	0	432	Cavalli et al., 2017 (24)
GSE109401	HuGene-2_0-st	19	5	24	Rivero-Hinojosa et al., 2018 (25)

**Table 2:** The characteristics of of MB patients in GSE85217 dataset.

Characteristics	Case	Proportion
**Age**		
0-3	62	14.35%
3-10	224	51.85%
10-17	105	24.31%
18+	41	9.49%
**Gender**		
Male	278	64.35%
Female	154	35.65%
**Histology**		
Classic	292	67.59%
Desmoplastic	69	15.97%
LCA	57	13.19%
MBEN	14	3.24%
**Subgroup**		
WNT	35	8.10%
SHH	122	28.24%
Group3	81	18.75%
Group4	194	44.91%
Metastasis		
M0	303	70.14%
M1	129	29.86%
**Status**		
Dead	118	27.31%
Alive	314	72.69%

LCA, large-cell anaplastic; MBEN, medulloblastoma with extensive nodularity.

### Prognostic analysis and gene set enrichment analysis (GSEA)

The prognostic role of TOP2A in MB was investigated in GSE85217 dataset. The survival curves of TOP2A were plotted by Kaplan–Meier survival analysis. The receiver operator characteristic (ROC) curves for TOP2A at 1, 3, and 5 years were constructed using the “survival ROC” package. A nomogram was constructed integrating several variables including gender, age, histology, met status, molecular subgroup and TOP2A expression, with a risk assessment model for 3- and 5-year survival rates. GSEA was performed though the Broad Institute GSEA version 4.1.0 software.

### Immunohistochemistry (IHC)

TOP2A IHC staining was conducted using formalin-fixed and paraffin-embedded MB samples, which were cut into 4 μm thick sections. Primary antibody against TOP2A (Proteintech Group, Chicago, IL, USA) was diluted at 1:100 and incubated with the MB sections at 4°C overnight. The active isoform of TOP2A stained in the nuclear were calculated (negative, score 0; < 1/3, score 1; 1/3–2/3, score 2; > 2/3, score 3).

### Cell culture and tissue collection

The MB cell lines Daoy and ONS-76 involved in this study was obtained from the Cell Resource Center, Peking Union Medical College (Beijing, China). Cells were maintained in 5% CO2 at 37°C in a humidified incubator. Dulbecco’s Modified Eagle’s Medium (Gibco, Grand Island, NY) supplemented with 10% FBS, 100 u/ml penicillin, and 100 u/ml streptomycin (Solarbio, Beijing, China) was used for the Daoy cell culture. Mycoplasma detection was performed on the cell line 1 month before the study. The MB cell line Daoy was authenticated using short tandem repeat (STR) profiling.

The MB tumor tissues and adjacent non-tumor brain tissues were obtained from patients who underwent intracranial space-occupying lesion resection in the Second Hospital of Hebei Medical University. None of the patients had undergone radiotherapy or chemotherapy before surgery. All the patients were diagnosed with MB based on postoperative pathology. The clinical information of all the patients enrolled in this study is summarised in **Supplementary Table 1**. The specimens were immediately placed in liquid nitrogen for snap freezing and then stored at – 80°C in a refrigerator. This study was approved by the Research Ethics Committee of The Second Hospital of Hebei Medical University. Written informed consent was acquired from all participants in the study.

### RNA extraction and RT-qPCR analysis

Total RNA was extracted from MB tissues or cells using TRIzol reagent (Invitrogen, Carlsbad, CA, USA) following the manufacturer’s instructions. The reaction system (20 ml) containing 1 mg total RNA was reverse-transcribed to cDNA using the RevertAid First Strand cDNA Synthesis Kit (Thermo Scientific, Waltham, MA, USA). The expression level of TOP2A was determined using All-in-One qPCR mix (FulenGene, Guangzhou, China), and the relative fold change was calculated by 2−ΔΔCt method. The amplification conditions comprised an initial pre-denaturation at 95°C for 10 min, followed by 40 cycles of denaturation at 95°C for 10 s, annealing at 60°C for 20 s, and 75°C for 15 s. GAPDH was used as the internal control. The primers used are shown in **Table 3**.

**Table 3:** Primers for RT-qPCR.

Gene	Primer direction	Sequence
TOP2A	PCR primer F	5’-ATCCTGCCAAAACCAAGAATCG-3’
	PCR primer R	5’-GTACAGATTTTGCCCGAGGAGC-3’
GAPDH	PCR primer F	5’-ATCCTGCCAAAACCAAGAATCG-3’
	PCR primer R	5’-GTACAGATTTTGCCCGAGGAGC-3’

### Plasmid and siRNA transfection

The coding sequence of TOP2A (NCBI Reference Sequence: NM_001067.4) was cloned into the pcDNA3.1 vector (Invitrogen) to achieve TOP2A overexpression. The pcDNA3.1 empty vector was used as negative control. Lipofectamine 2000 Reagent (Invitrogen) was used for plasmid transfection. Three small interfering RNAs (siRNAs) specifically targeting TOP2A were synthesised by GenePharma (Suzhou, Jiangsu, China). Daoy cells were transfected with 30 nM siRNAs using Lipofectamine RNAiMAX Reagent (Invitrogen), and knockdown efficiencies were measured by RT-PCR and western blot analyzes. The sequences of the siRNAs and the negative control (NC) are presented in **Table 4**.

**Table 4:** Sequences of siRNAs.

Name	Nucleotide sequence	
Si-TOP2A -1	Sense	5’-GGUCAGAAGAGCAUAUGAUTT-3’
	Antisense	5’-AUCAUAUGCUGUUCUGACCTT-3’
Si-TOP2A -2	Sense	5’-GACCAACCUUCAACYAUCUTT-3’
	Antisense	5’-AGAUAGUUGAAGGUUGGUCTT-3’
Si-TOP2A -3	Sense	5’-GGUGAGAUGUCACUAAUGATT-3’
	Antisense	5’-UCAUUAGUGACAUCUCACCTT-3’
Negative control (NC)	Sense	5’-UUCUCCGAACGUGUCACGUTT-3’
	Antisense	5’-ACGUGACACGUUCGGAGAATT-3

### Western blot analysis

Total protein from MB cells was extracted using Radio-Immunoprecipitation Assay buffer containing a protease inhibitor cocktail and phosphatase inhibitor cocktail (Solarbio). Protein concentrations were determined using a BCA assay kit (Solarbio), and 50 μg of protein from each group was separated using SDS-PAGE and transferred to a PVDF membrane (Millipore, Billerica, MA, USA). The antibodies used in the western blot assay were TOP2A (Proteintech Group, Chicago, IL, USA), β-catenin (Proteintech Group), and GAPDH (Affinity, Cincinnati, OH, USA).

### CCK-8 assay

MB cell suspensions were seeded into 96 well plates at a density of 2 × 10^3^ cells/well. Twenty-four hours after siTOP2A transfection, cells in the irradiation (IR) group were exposed to 8 Gy IR (28) with a 6 MV X-ray beam from an Elekta linear accelerator (Synergy S; Elekta Instrument AB, Stockholm, Sweden) at a dose rate of 300 cGy/min, whereas cells in the non-irradiation group were not exposed to IR. Cell Counting Kit-8 (CCK-8) reagent (Dojindo Molecular Technologies, Inc., Kumamoto, Japan) was added to the cell medium at 10 μl/well every day for 7 days. After adding CCK-8 for 1 h, the absorbance of each well at 450nm was measured using the Multiskan Spectrum (Thermo Fisher, Rockford, IL, USA).

### Wound healing assay

After transfected for 24h, the MB cells in the irradiation group were treated with 8 Gy IR. The MB cells were plated into six−well plates at a density of 3 × 10^5^ cells/well. Until a confluent cell monolayer was formed, an artificial wound was induced in the middle of each monolayer by using a 10 μl pipette tip. Migrated cells were visualised by taking pictures at fixed positions in the epithelial wound at 0, 6, 12, and 24 h post-injury using a light microscope (Carl Zeiss AG, Dublin, CA, USA).

### Transwell invasion and migration assays

Transwell assays were performed using Transwell Permeable Supports (8.0 um; Costar 3422; Corning, NY, USA) to evaluate the migration and invasion abilities of transfected MB cells with or without exposure to 8 Gy IR. For the invasion assay, 1 × 10^5^ cells were seeded into the upper chamber in serum-free medium with Matrigel Matrix (BD Biosciences, San Jose, CA, USA) precoated inserts, whereas in the migration experiment, the cells were seeded onto membranes without Matrigel Matrix. Medium containing 10% serum was added to the bottom of the well, and, it served as a chemoattractant. After 24 h of incubation, the cells that had invaded through the foramen were fixed with methanol for 15 min and stained with 0.1% crystal violet (Solarbio) for 30 min. Ten random fields of invading cells from each chamber were photographed and counted under a light microscope (Carl Zeiss AG).

### Colony formation assay

Colony formation assays were performed to evaluate the radiosensitivity of MB cells after TOP2A knockdown. Transfected MB cell suspensions containing 200, 300, 500, 800, 2000, and 3000 cells were seeded into six-well plates and individually treated with different doses of IR (0, 2, 4, 6, 8 and 10 Gy). The cells were then cultured for another 2 weeks until colonies were formed. Colonies were fixed with methanol for 15 min and stained with 0.1% crystal violet for 15 min. Colonies visible to the eyes (more than 50 cells) were counted under a light microscope (Carl Zeiss AG). The survival curves of the clone formation were analyzed though the single-hit multi-targeted model (y = 1-(1-exp(−k*x))ˆN) with GraphPad Prism 6.0 software. The ‘y’ represents the cell survival fraction (SF), which was calculated according to the following formula: SF = colony numbers formed in experimental group/[plating cell number × plating efficiency (PE)]; PE = (colony number in the control group/plating cell number in the control group) × 100%. The ‘x’ represents each radiation dose. Through the survival curves formed by the single-hit multi-targeted model, the k and extrapolation number (N) values were obtained. D_0_ (mean lethal dose) = 1/k, Dq (quasi-threshold dose) = D_0_ × ln (N), and SF_2_ (survival fraction after 2 Gy IR) = the colony number formed after 2 Gy IR/[plating cell number × PE].

### Statistical analysis

The R software (Version 4.1.2) and GraphPad Prism software (version 6.0) were used for the statistical analysis and chart drawing. The differences of TOP2A expression in GEO datasets were analyzed using the Wilcoxon signed-rank test. The differences in expression between MB and matched normal brain tissues were analyzed by a paired samples t test. Survival analyzes were conducted using Kaplan-Meier analysis methods *via* the “survival” and “survminer” R packages. A nomogram was constructed *via* “rms” package of R. Changes in proliferation, migration, and invasion after transfection and irradiation were analyzed using the independent samples t test. Each experiment independently repeated in triplicate. All data were presented as the mean ± SD. Statistical significance was set at P<0.05.

## Results

### TOP2A is upregulated in MB

**Figure 1:**
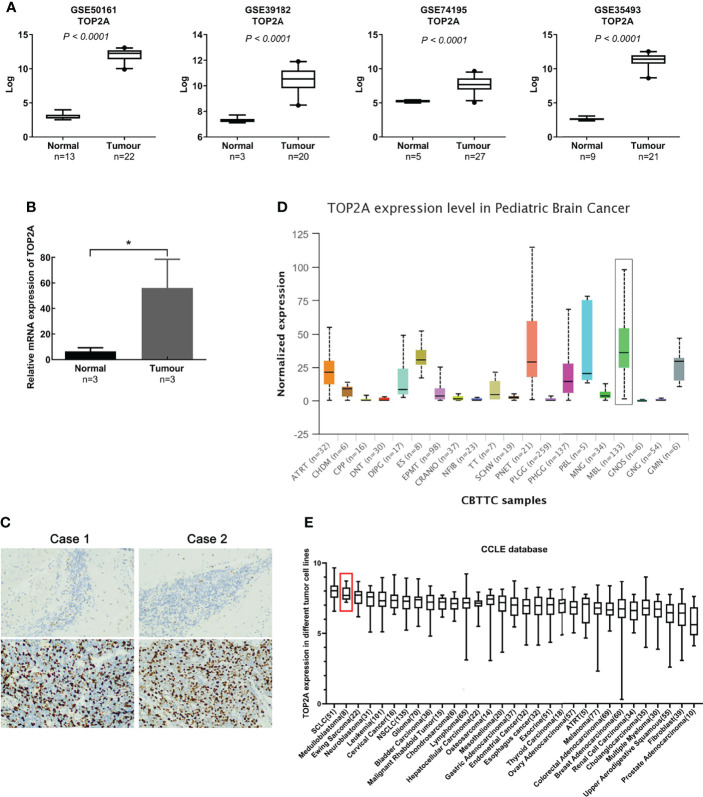
TOP2A is high expressed in MB. **(A)** The expression of TOP2A in normal brain tissues and MB tissues was analyzed according to microarray data (GSE50161, GSE39182, GSE74195 and GSE35493). Values are the median with 95% CI. **(B)** The expression of TOP2A in 3 pairs of MB tissues and corresponding normal brain tissues was analyzed through RT-qPCR analysis. Transcript levels were normalized to GAPDH expression. *P<0.05. **(C)** The expression of TOP2A in MB tissues and adjacent normal brain tissues was detected through IHC (magnification × 200 in each picture). **(D)** The expression of TOP2A was assessed in 20 types of paediatric brain tumors with UALCAN, including MB (indicated by a black box). **(E)** The expression of TOP2A across various tumor cell lines, including MB cell lines (indicated by a red box), is shown in the CCLE database.

Gene expression profiles of MB (GSE50161, GSE39182, GSE74195 and GSE35493) were acquired from the GEO database. Analysis of the four gene expression datasets showed that TOP2A was significantly upregulated in MB than in normal brain samples (**Figure 1A**). Subsequently, RT-qPCR was used to detect the expression of TOP2A in three cases of MB tissues and adjacent normal brain tissues. The results showed that the expression of TOP2A in tumor tissues was significantly higher than that in adjacent normal tissues (**Figure 1B**), which was consistent with the results of the four GEO datasets. Moreover, immunohistochemistry was performed to detect TOP2A expression, and the results demonstrated that the stain intensity of TOP2A was obviously stronger in MB tissues than that in adjacent normal brain tissues (**Figure 1C**). We also assessed the expression level of TOP2A in 20 types of paediatric brain tumors with UALCAN, and TOP2A expression in MB ranked first among these tumors (**Figure 1D** and **Supplementary Table 2**). Furthermore, the CCLE database was used to assess TOP2A expression in a variety of tumor cell lines, and the expression level of TOP2A in MB ranked second among these tumor cell lines (**Figure 1E**).

### Correlations between TOP2A expression and clinicopathologic characteristics

**Figure 2:**
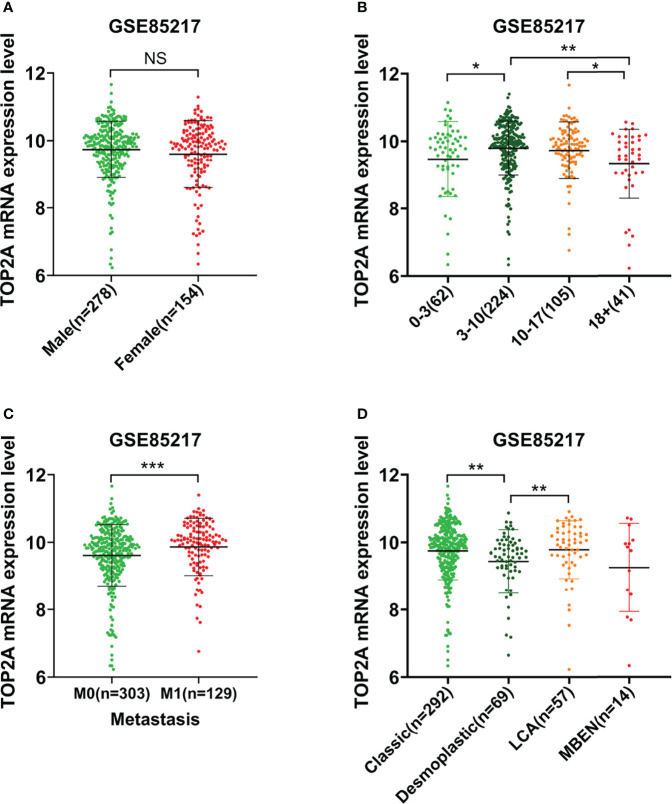
The relationship between the expression level of TOP2A and clinicopathological features in GSE85217 dataset. **(A)** The expression level of TOP2A in different genders. **(B)** The expression level of TOP2A in different age groups. **(C)** The expression level of TOP2A in different metastatic status. **(D)** The expression level of TOP2A in different histology types.

The relationship between TOP2A expression and clinical traits was analyzed in GSE85217 database. Results showed that there was nonsignificant correlation between TOP2A expression and gender (**Figure 2A**). For different age groups, patients in 3-10 years and 10-17 years groups exhibited higher levels of TOP2A expression than 0-3 years and 18+ years groups (**Figure 2B**). Moreover, the expression level of TOP2A was significantly increased in metastatic group compared with non-metastatic group (**Figure 2C**). In addition, the expression patterns of TOP2A based on MB histology type was shown in **Figure 2D**, and TOP2A expressed relatively higher in classic and LCA subtypes than in desmoplastic and MBEN subtypes.

### TOP2A acts as a prognostic marker of MB

**Figure 3:**
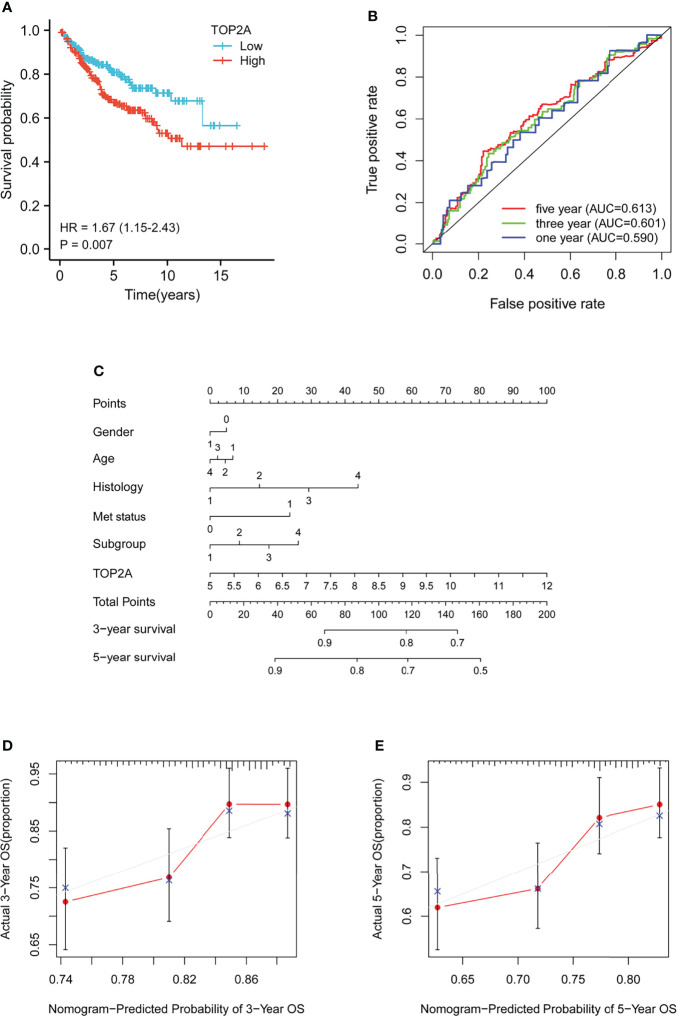
Prognostic analysis of TOP2A in MB patients in GSE85217 dataset. **(A)** Kaplan-Meier survival curves of the high and low TOP2A expression groups divided by the median score. **(B)** ROC curve analysis of the prognostic value of TOP2A for MB patients. **(C)** Nomogram for predicting 3- and 5-year survival rates of MB patients. The variables include gender (0, female; 1, male), age (1, 0-3 years; 2, 3-10 years; 3, 10-17 years; 4, 18+ years), histology (1, classic; 2, desmoplastic; 3, LCA; 4, MBEN), met status (0, M0; 1, M1), molecular subgroup (1, WNT; 2, SHH; 3, Group3; 4, Group4) and TOP2A expression, which were integrated in the nomogram. **(D, E)** The calibration curves for predicting survival probability at 3 and 5 years.

Kaplan-Meier survival curves was plotted to calculate the survival probability of high and low TOP2A expression groups divided by the median score. As shown in **Figure 3A**, high expression of TOP2A related to remarkably poor survival of MB patients in comparison with the low expression group in GSE85217 dataset. The prognostic value of TOP2A expression in MB was also assessed using ROC curve analysis. The values of area under curve for 1, 3, and 5 years were 0.590, 0.601, and 0.613, which indicated that TOP2A might serve as a predictive marker of survival (**Figures 3B**). To further investigate the prognostic value of TOP2A in MB, a nomogram was constructed with a risk assessment model for 3- and 5-year survival rates based on GSE85217 datasets. The nomogram integrated several variables including gender, age, histology, met status, molecular subgroup and TOP2A expression, and it revealed that the TOP2A expression level was the leading factor for predicting nomogram (**Figure 3C**). The calibration curves for the survival probability at 3 and 5 years showed that the prediction by nomogram is matched well with the actual observations (**Figures 3D, E**).

### Overexpression of TOP2A promotes MB cell proliferation and invasion

**Figure 4:**
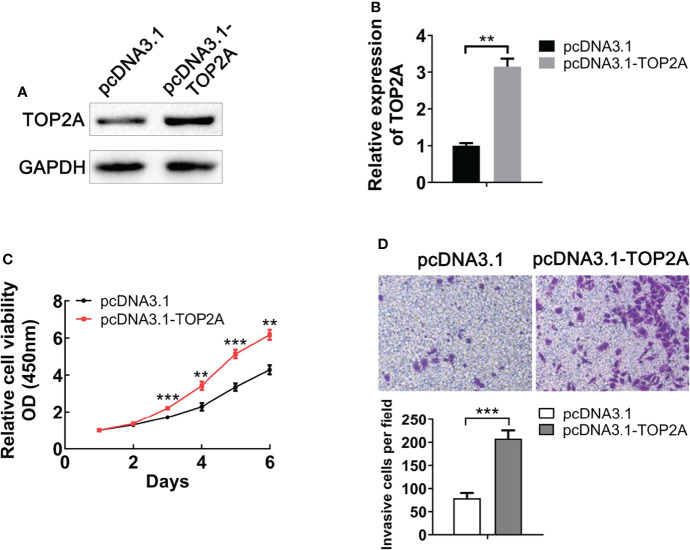
Upregulation of TOP2A promotes MB cell proliferation and invasion. **(A)** The expression of TOP2A was detected by western blot analysis after transfecting Daoy cells with pcDNA3.1-TOP2A. **(B)** Semi-quantitative densitometric analysis was used to measure the relative levels of TOP2A. **P<0.01. **(C)** CCK-8 assay was used to detect the proliferation ability of pcDNA3.1-TOP2A-transfected Daoy cells. **P<0.01, ***P<0.001. **(D)** Transwell assays precoated with Matrigel Matrix were performed to assess the invasive ability of pcDNA3.1-TOP2A-transfected Daoy cells. ***P<0.001.

To evaluate the functional role of TOP2A on MB, we upregulated its expression by constructing pcDNA3.1-TOP2A vector and transfecting it into Daoy cells. Western blot analysis demonstrated that the expression of TOP2A was significantly increased in pcDNA3.1-TOP2A transfected group than the pcDNA3.1 empty vector transfected group (**Figures 4A, B**). Growth curves drawn by CCK8 analysis revealed that overexpression of TOP2A drastically promoted MB cell proliferation (**Figure 4C**). Moreover, transwell assays showed that the invasive capacity of MB cells was significantly increased following TOP2A overexpression (**Figure 4D**). These data indicate that upregulation of TOP2A enhances the proliferative and invasive ability of MB cells.

### Knockdown of TOP2A inhibits MB cell proliferation

**Figure 5:**
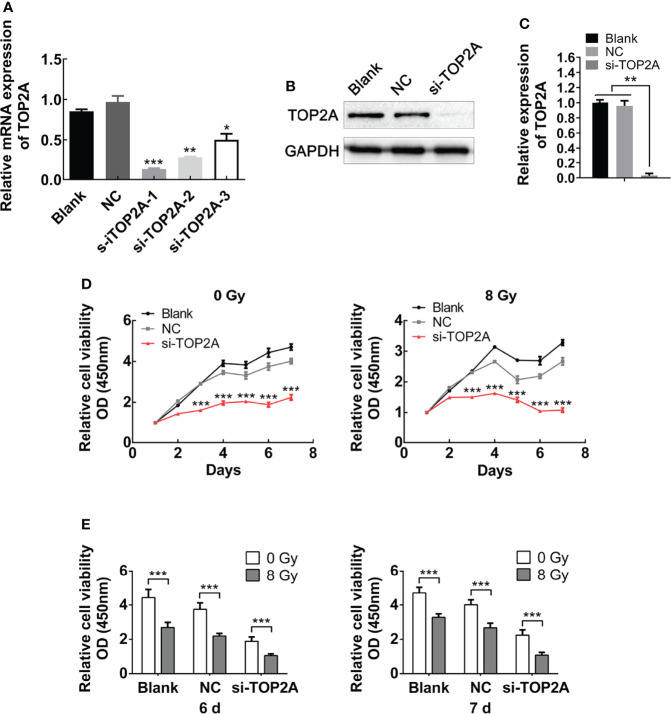
Downregulation of TOP2A inhibits MB cell proliferation. **(A)** The expression of TOP2A was detected by RT-qPCR after transfecting Daoy cells with TOP2A siRNAs. *P<0.05, **P<0.01, ***P<0.001. **(B)** The expression of TOP2A was detected by western blot analysis after transfecting Daoy cells with si-TOP2A. **(C)** Semi-quantitative densitometric analysis was used to measure the relative levels of TOP2A. **P<0.01. **(D)** CCK-8 assay was used to detect the proliferation ability of si-TOP2A-transfected MB cells with or without exposure to 8 Gy IR. ***P<0.001. **(E)** The relative proliferation of si-TOP2A-transfected MB cells was shown at days 6 and 7 post IR treatment. ***P<0.001.

For further validation of the function of TOP2A in MB cells, three different siRNAs against TOP2A (si-TOP2A-1, si-TOP2A-2, and si-TOP2A-3) were synthesised and transfected into Daoy cells, and 48 h later, the knockdown efficiency was examined by RT-qPCR. The results revealed that all three siRNAs effectively inhibited the expression of TOP2A, and si-TOP2A-1 had the highest knockdown efficiency (**Figure 5A**). Moreover, western blot analysis indicated that the expression of TOP2A in Daoy and ONS-76 cells was drastically decreased following si-TOP2A-1 transfection (**Figures 5B, C** and **Supplementary Figures 2A, B**). Therefore, si-TOP2A-1, later referred to as si-TOP2A, was selected for follow-up experiments. CCK-8 proliferation assay was used to assess the growth curves of si-TOP2A-transfected MB cells with or without exposure to 8 Gy IR. The results showed that TOP2A downregulation inhibited the proliferative capacity of Daoy and ONS-76 cells, whereas TOP2A knockdown in combination with IR significantly reduced the growth of Daoy and ONS-76 cells compared with those treated with si-TOP2A alone (**Figures 5D, E**, **Supplementary Figures 1** and **2C, D**).

### Knockdown of TOP2A inhibits MB cell migration and invasion, and sensitises tumor cells to radiotherapy

**Figure 6:**
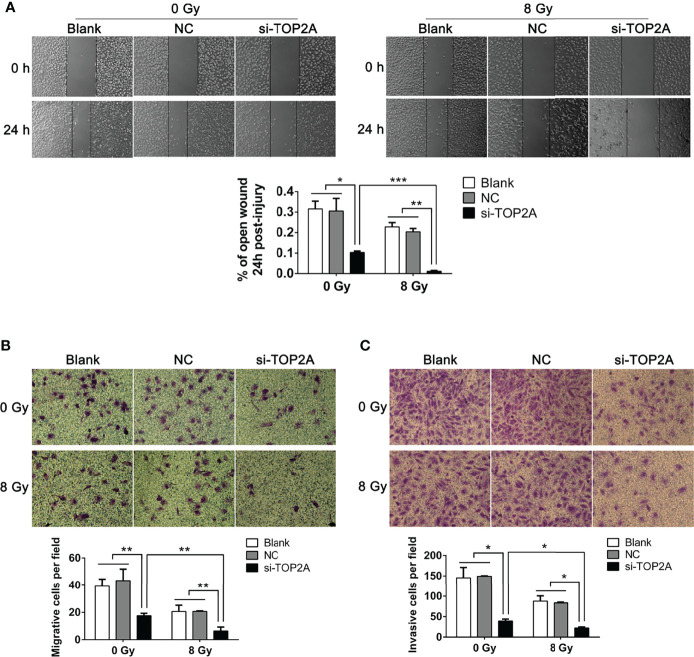
Downregulation of TOP2A inhibits MB cell migration and invasion. **(A)** Wound healing assays were performed to evaluate the migratory ability of si-TOP2A-transfected MB cells with or without exposure to 8 Gy IR. *P<0.05, **P<0.01, ***P<0.001. **(B)** Transwell assays were performed to assess the migratory ability of si-TOP2A-transfected MB cells with or without exposure to 8 Gy IR. **P<0.01. **(C)** Transwell assays precoated with Matrigel Matrix were performed to assess the invasive ability of si-TOP2A-transfected MB cells with or without exposure to 8 Gy IR. *P<0.05.

The migration and invasion abilities of tumor cells are closely related to their metastasis. The effect of TOP2A on MB cell migration was detected by wound healing assay and transwell migration assay. There was a significant decrease in cell migration during the closure of the artificial wound created in the confluent cell monolayer after TOP2A downregulation, as shown by the wound healing assay, and subsequent IR exposure further enhanced the inhibitory effect of si-TOP2A on cell migration (**Figure 6A**). Similarly, the transwell migration assay demonstrated that the number of cells invading through the foramen was fewer in the TOP2A knockdown groups than that in the control group, and si-TOP2A transfection in combination with IR significantly reduced the number of migratory cells compared with TOP2A knockdown alone (**Figure 6B**). Additionally, the transwell invasion assay showed that the number of cells invading through the foramen was decreased by TOP2A knockdown alone, and the invasive ability of MB cells was further significantly decreased by TOP2A knockdown in combination with IR (**Figure 6C** and **Supplementary Figure 2E**). Taken together, these data indicate that si-TOP2A transfection in combination with IR reduced the migration and invasion ability of Daoy cells, which suggests that TOP2A knockdown may sensitize MB cells to radiotherapy.

### Downregulation of TOP2A reduces radioresistance of MB cells

**Figure 7:**
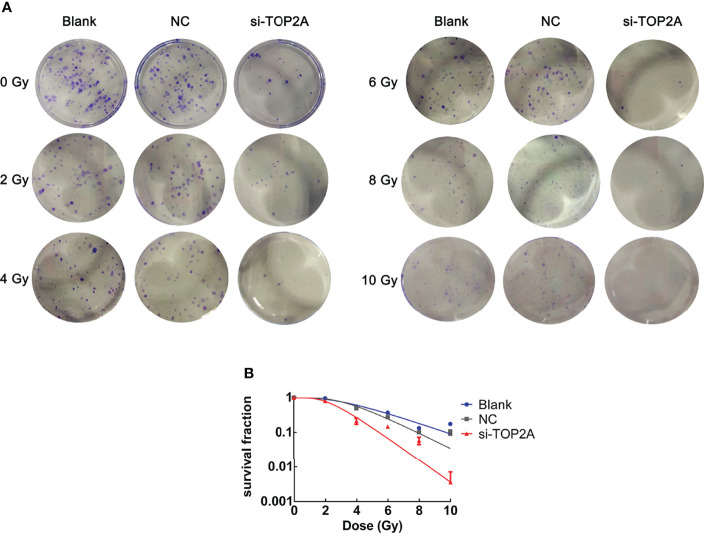
Downregulation of TOP2A limits the radioresistance of MB cells. **(A)** Colony formation assays were employed to evaluate the radiosensitivity of MB cells individually exposed to 0, 2, 4, 6, 8, and 10 Gy IR, respectively. **(B)** Cell survival curves analysis were performed to assess the radioresistance of MB cells.

To determine whether TOP2A knockdown could reduce the radioresistance of MB cells, colony formation assays were conducted, and different groups were individually exposed to IR doses of 0, 2, 4, 6, 8, and 10 Gy. The results showed that downregulation of TOP2A significantly decreased colony numbers compared with the control groups after IR treatment (**Figure 7A**). Cell survival curve analysis revealed that the fraction of surviving MB cells was significantly reduced upon TOP2A downregulation. Compared with the control cells, si-TOP2A-transfected cells had decreased D_0_, Dq and SF_2_ values (**Figure 7B** and **Table 5**), which indicated that TOP2A knockdown suppressed resistance to radiotherapy in MB cells.

**Table 5:** Parameters of radiosensitivity in different groups of Daoy cells.

	D_0_	Dq	SF_2_
**Blank**	2.39 ± 0.34	3.50 ± 0.34	1.00 ± 0.16
**NC**	2.40 ± 0.35	3.11 ± 0.05	1.01 ± 0.24
**si-TOP2A**	1.20 ± 0.28	2.69 ± 0.35	0.80 ± 0.23

### TOP2A affects the activity of Wnt/β-catenin signaling pathway

**Figure 8:**
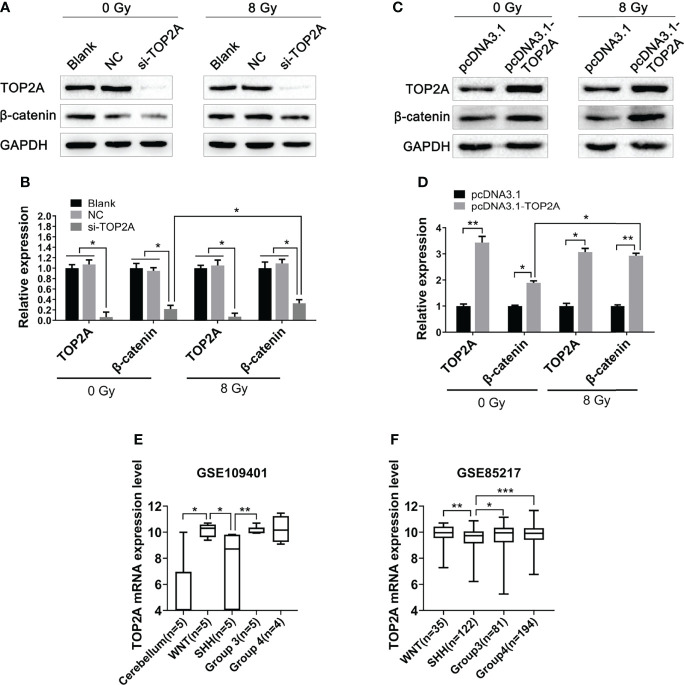
Downregulation of TOP2A inhibits the activity of the Wnt/β-catenin signaling pathway. **(A)** Western blot assay was used to detect the expression of β-catenin following TOP2A knockdown alone or combined treated with 8 Gy IR. **(B)** Semi-quantitative densitometric analysis was used to measure the relative levels of TOP2A and β-catenin following TOP2A knockdown alone or combined treated with 8 Gy IR. *P<0.05. **(C)** Western blot assay was used to detect the expression of β-catenin following TOP2A overexpression alone or combined treated with 8 Gy IR. **(D)** Semi-quantitative densitometric analysis was used to measure the relative levels of TOP2A and β-catenin following TOP2A overexpression alone or combined treated with 8 Gy IR. *P<0.05, **P<0.01. **(E)** The expression of TOP2A in cerebellum tissues and MB molecular subgroups was analyzed in GSE109401. *P<0.05, **P<0.01. **(F)** The expression of TOP2A in MB molecular subgroups was analyzed in GSE85217. **P<0.01, ***P<0.001.

The Wnt/β-catenin signaling pathway is involved in the occurrence and development of various tumors, and is closely related to tumor proliferation and migration (29). Our previous study has found that this pathway is closely related to radioresistance in oesophageal cancer (30). In pancreatic cancer, the Wnt/β-catenin signaling pathway is activated by high TOP2A expression (31). Therefore, in the present study, we investigated whether TOP2A affects the radioresistance of MB cells through the Wnt/β-catenin signaling pathway. Western blot analysis was used to detect the expression of key proteins in this pathway following TOP2A knockdown alone or in combination with 8 Gy IR in Daoy and ONS-76 cells. The results showed that the expression of β-catenin was significantly decreased after TOP2A downregulation, and si-TOP2A transfection in combination with IR treatment increased TOP2A expression compared with si-TOP2A transfection alone (**Figures 8A, B** and **Supplementary Figures 3A, B**). Moreover, upregulation of TOP2A by transfected Daoy cells with pcDNA3.1-TOP2A plasmid significantly enhanced the expression of β-catenin, and pcDNA3.1-TOP2A transfection in combination with IR treatment increased TOP2A expression compared with pcDNA3.1-TOP2A transfection alone (**Figures 8C, D**). Collectively, these data indicate that downregulation of TOP2A reduces the tumorigenicity and radioresistance of MB cells may through inhibiting the activity of Wnt/β-catenin signaling pathway.

Because the WNT subgroup of MB is characterized by aberrant WNT signaling with CTNNB1 mutation, we speculated weather TOP2A is expressed differently among different molecular subtypes of MB. Data analysis of GSE109401 showed that the expression of TOP2A was significantly higher in the WNT group than in the normal cerebellum tissues. In the SHH group and non-WNT/non-SHH groups, TOP2A expression was higher than that in the cerebellum, but there was nonsignificant difference, which may be due to the limitation of the sample size. The expression of TOP2A in the WNT group and group 3 was significantly higher than that in the SHH group (**Figure 8E**). We then analyzed the expression of TOP2A in GSE85217, which consisted of 763 primary medulloblastoma samples. The results demonstrated that the expression of TOP2A is remarkably upregulated in the WNT group, group 3, and group 4 relative to the SHH group, and there was no obvious difference among WNT, group 3, and group 4 (**Figure 8F**).

### Wnt/β-catenin signaling pathway is enriched in TOP2A high-expression phenotypes

**Figure 9:**
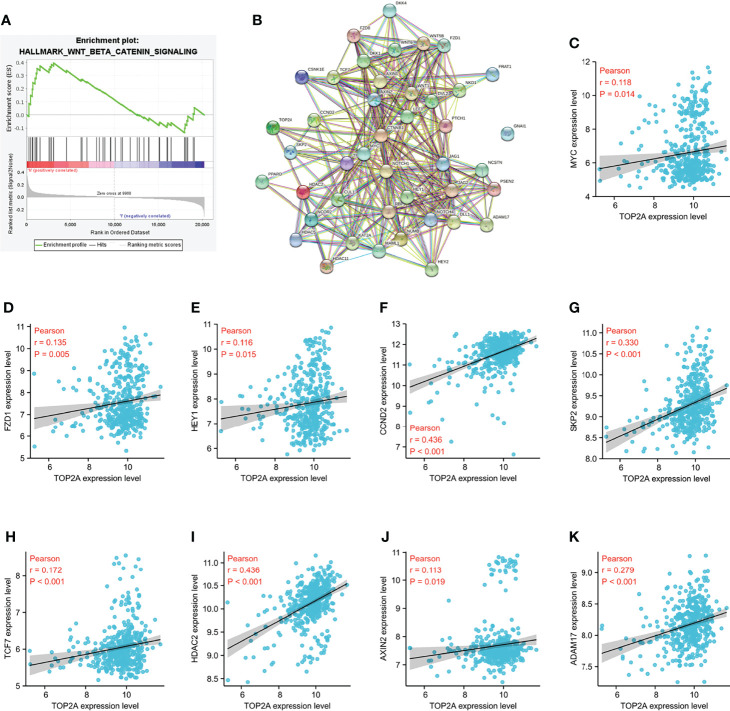
Enrichment pathways analysis of TOP2A based on GSE85217 dataset. **(A)** HALLMARK_WNT_BETA_CATENIN signaling pathways enriched across DEGs by GSEA analysis. **(B)** PPI network of the genes of Wnt/β-catenin signaling pathway in GSEA analysis constructed by STRING analysis. **(C K)** Co-expression analysis of TOP2A with the core genes of Wnt/β-catenin signaling pathway.

Gene set enrichment analysis (GSEA) was performed to identify the signaling pathways enriched between high and low TOP2A expression groups. Consist with the results of western blot analysis, the DEGs were also markedly involved in HALLMARK_WNT_BETA_CATENIN signaling pathways in TOP2A high-expression phenotypes (NES = 1.09, **Figure 9A**). The PPI network of TOP2A and the genes of Wnt/β-catenin signaling pathway in GSEA analysis was constructed by STRING analysis (**Figure 9B**). Then the relationship of TOP2A and the core genes of Wnt/β-catenin signaling pathway was investigated through gene co-expression analysis. The results showed that the expression of TOP2A was significantly positively correlated with MYC, FZD1, HEY1, CCND2, SKP2, TCF7, HDAC2, AXIN2, ADAM17 (**Figures 9C–K**).

## Discussion

Medulloblastoma is one of the most malignant neuroepithelial tumors of the central nervous system. It grows rapidly, is difficult to resect, and spreads easily through the cerebrospinal fluid (32). With a deep understanding of MB and the progress of related clinical technology, the current therapeutic schedule for MB is postoperative radiotherapy and chemotherapy based on risk stratifications (33). Although the whole central nervous system radiotherapy may result in a series of short-term and long-term reactions, such as brain oedema, elevated intracranial pressure, hearing loss, hypothyroidism, and cognitive dysfunction, radiotherapy remains crucial in the treatment of MB (34–36). Radioresistance seriously restricts the therapeutic effect in MB patients, thus, investigating the related molecular mechanism of radioresistance and reducing its occurrence during the process of radiotherapy are tasks of substantial significance.

Uncontrolled proliferation of tumor is closely related to various abnormal signaling pathways in the body. As a key catalytic enzyme in the initiation of DNA replication, TOP2A has been widely recognised as a potential target for cancer therapy. It is closely related to the prognosis and recurrence of, for example, breast cancer, lung cancer, and peripheral T-cell lymphoma (37–39). The main target of some chemotherapeutic drugs, such as etoposide, is TOP2A. TOP2A gene mutations or fusions can lead to chemotherapy drug tolerance (40, 41). Many studies have suggested that TOP2A is upregulated in various tumors, including MB, and our study confirmed that TOP2A is highly expressed in MB. We downloaded four gene expression profiles (GSE50161, GSE39182, GSE74195 and GSE35493) from the GEO database, and data analysis results showed that TOP2A was significantly highly expressed in MB than in normal brain samples. Consistent with the results of the GEO datasets, RT-qPCR also revealed that the expression of TOP2A in MB tissues was significantly higher than that in adjacent normal tissues. Moreover, high expression of TOP2A related to remarkably poor survival, and act as a prognostic marker of MB patients. In addition, Deguchi and Song found that TOP2A is involved in the proliferation of glioma through lncRNA ECONEXIN and miR-144-3p (42, 43), but the relationship between TOP2A and proliferation, migration and invasion of MB remains unclear. In this study, CCK-8 assay was used to detect the effect of TOP2A on the proliferation of MB cells, and the proliferative capacity of MB cells obviously decreased when TOP2A was knocked down. Through wound healing assay and transwell assay, we found that the migration and invasion ability of MB cells decreased significantly after knockdown of TOP2A. On the other hand, overexpression of TOP2A enhance the proliferation and invision of MB cell. Taken together, these data suggested that TOP2A may play a key role in promoting multiple oncogenic properties in MB and the upregulation of TOP2A in MB may contribute to tumor progression.

DNA DSBs cause instability in the human genome, and topoisomerase II (TOP2) participates in secondary structure-mediated DNA fragility (44). Several studies have reported that TOP2A exerts crucial role in radiation-induced chromatid breaks in DSBs and is closely related to the effects of radiotherapy (15–17). In the present study, si-TOP2A-transfected MB cells were exposed to 8 Gy IR, and the results showed that si-TOP2A transfection in combination with IR exposure obviously reduced the proliferation, migration and invasion ability of Daoy cells compared with those transfected with si-TOP2A alone, which suggests that TOP2A knockdown may increase the radiosensitivity of MB cells and sensitize MB cells to radiotherapy. Moreover, the cell survival curve was obtained by colony formation assay, and radiosensitivity parameters, such as D_0_, Dq, and SF_2_, were calculated. Upon TOP2A downregulation, the survival fraction of MB cells was significantly reduced, and the D_0_, Dq, and SF_2_ values were obviously decreased, indicating that TOP2A knockdown could decrease the radioresistance of MB cells.

The aberrant Wnt/β-catenin signaling pathway is closely correlated with the initiation and development of various types of tumors, and plays a crucial role in tumorigenesis and therapeutic response (45–47). Monica et al. found that the Wnt/β-catenin signaling pathway is involved in the development of radioresistance in prostate cancer (48). Our prior study found that the ectopic subcellular localisation of NRAGE could induce β-catenin translocation into the nucleus and promote radioresistance in oesophageal squamous cell carcinoma (30). Pei et al. found that in pancreatic cancer, the Wnt/β-catenin signaling pathway could be activated by the high expression of TOP2A, which then promotes tumor proliferation and invasion (31). In our present study, the relationship between TOP2A and the Wnt/β-catenin signaling pathway was analyzed. Western blot analysis showed that the expression of β-catenin was significantly decreased following si-TOP2A transfection alone or in combination with RT, and GSEA analysis revealed that Wnt/β-catenin signaling pathway is enriched in TOP2A high-expression phenotypes, indicating that knockdown of TOP2A may inhibit the activity of the Wnt/β-catenin signaling pathway, thus reduce the tumorigenicity and radioresistance of MB cells.

We analyzed TOP2A expression in different molecular subtypes of MB based on GEO datasets, and the results revealed that the expression of TOP2A was significantly higher in the WNT and non-WNT/non-SHH groups than in the SHH group. Previous studies have reported that activation of the Wnt/β-catenin signaling pathway contributes to radioresistance in most tumor types, including glioma, hepatocellular carcinoma, and nasopharyngeal carcinoma (49–51). However, radiation resistance among the different molecular subtypes of MB has rarely been reported. For the WNT subgroup of MB exhibited activated WNT signaling with CTNNB1 mutation, and had a relatively higher level of TOP2A, we speculated that its resistance to radiation may be higher than that of other molecular subtypes, and we plan to conduct that investigation in further research.

In conclusion, our study is the first to explore the effect of TOP2A on MB radiotherapy. TOP2A was highly expressed in MB, and when the expression of TOP2A was downregulated, the proliferation, migration, and invasion abilities of MB cells decreased significantly. Additionally, knockdown of TOP2A reduced the radioresistance of MB cells. The inhibitory effects of si-TOP2A on cell proliferation, survival, migration, and invasiveness and radioresistance of MB cells may be mediated through inhibition of the the activity of Wnt/β-catenin signaling pathway; however, the specific molecular mechanism requires further research. Our findings are helpful to better understanding of the radioresistance of MB and suggest that targeting TOP2A may be a therapeutic strategy for MB radiotherapy in the future.

## Data availability statement

The original contributions presented in the study are included in the article/**supplementary material**. Further inquiries can be directed to the corresponding authors.

## Ethics statement

The studies involving human participants were reviewed and approved by the research ethics committee of The Second Hospital of Hebei Medical University. Written informed consent to participate in this study was provided by the participants’ legal guardian/next of kin.

## Author contributions

YZ, HY, and LW carried out the experiments and wrote the manuscript. HZ analyzed the data. GZ and ZX performed cell culture and cell irradiation. XX designed the experimental and reviewed the manuscript. All authors contributed to the article and approved the submitted version.

## Funding

This study was supported by the Scientific Research Foundation from Public Health Department of Hebei province, China (20170556 to YZ) and the National Natural Science Foundation of China (81702299 to HY).

## Conflict of interest

The authors declare that the research was conducted in the absence of any commercial or financial relationships that could be construed as a potential conflict of interest.

## Publisher’s note

All claims expressed in this article are solely those of the authors and do not necessarily represent those of their affiliated organizations, or those of the publisher, the editors and the reviewers. Any product that may be evaluated in this article, or claim that may be made by its manufacturer, is not guaranteed or endorsed by the publisher.
