# Supplementary figures and images for: TOP2A correlates with poor prognosis and affects radioresistance of medulloblastoma
Source: Front Oncol. 2022 Jul 15;12:918959. doi: 10.3389/fonc.2022.918959 (PMC9337862; doi:10.3389/fonc.2022.918959)

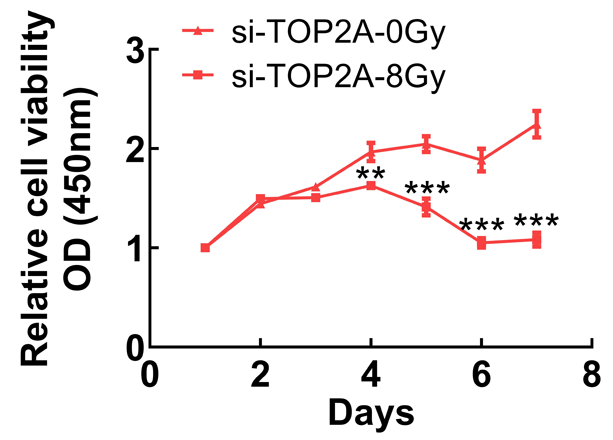

Supplement: Supplementary file 1 [file Image_1.tif]

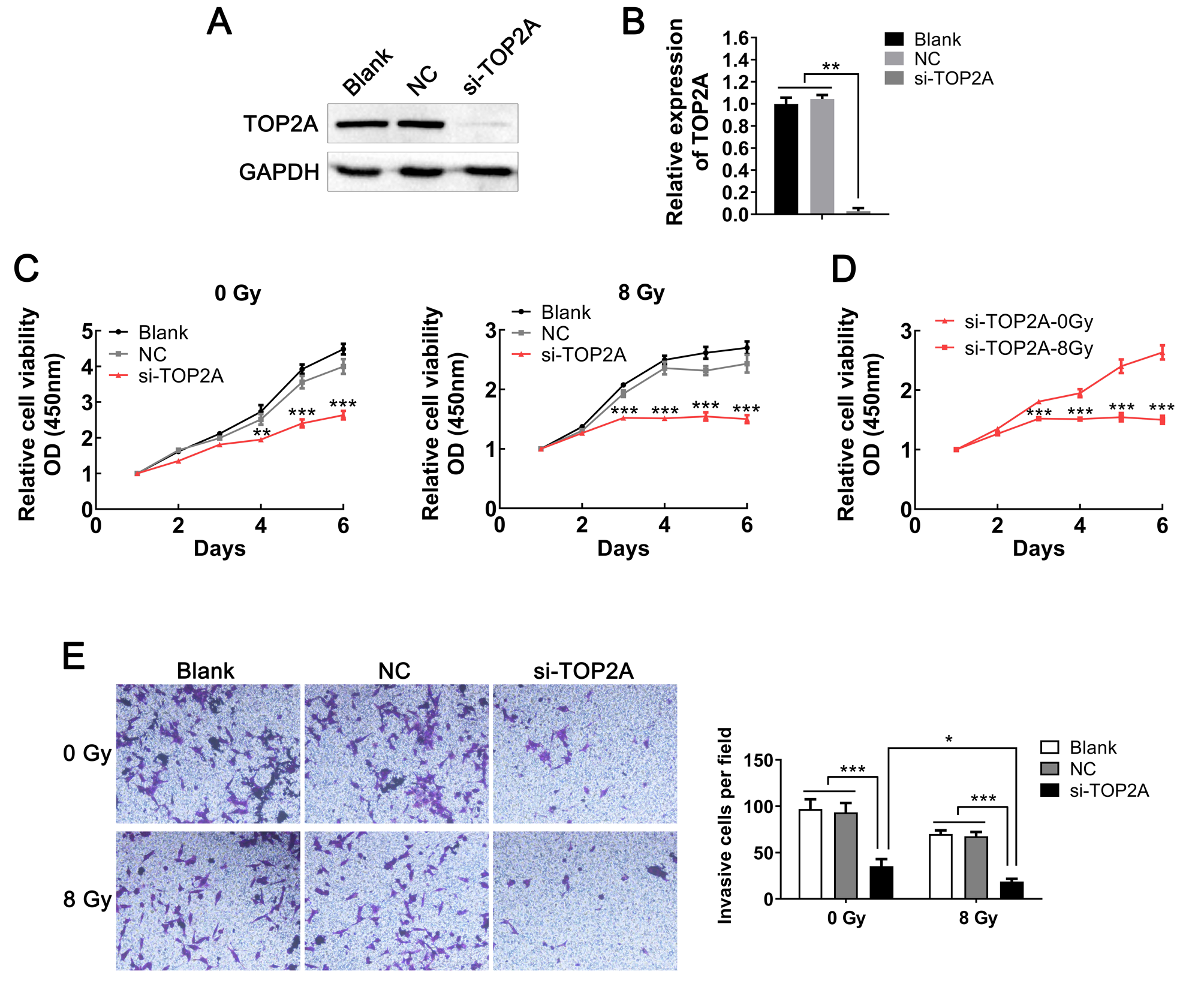

Supplement: Supplementary file 2 [file Image_2.tif]

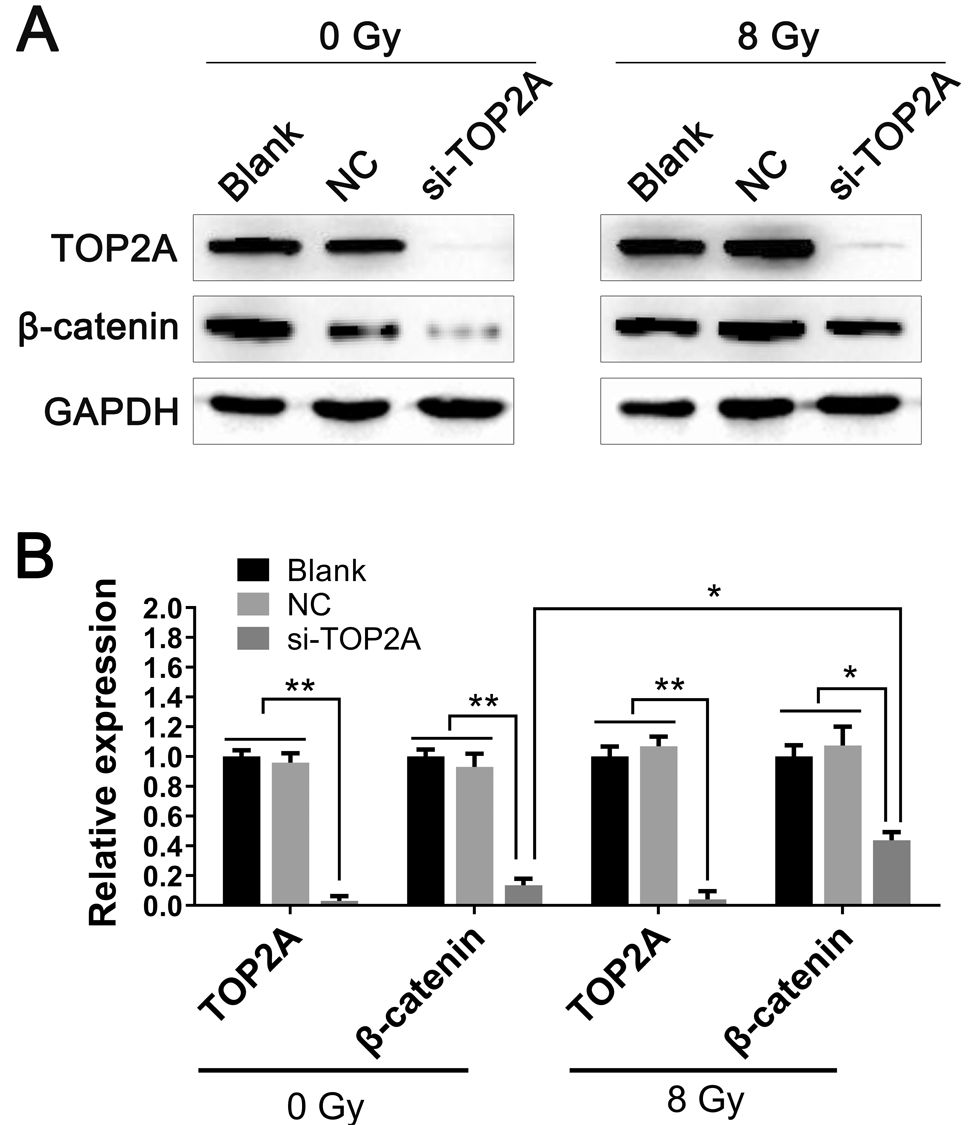

Supplement: Supplementary file 3 [file Image_3.tif]
