# Supplementary material for: TOP2A correlates with poor prognosis and affects radioresistance of medulloblastoma
Source: Front Oncol. 2022 Jul 15;12:918959. doi: 10.3389/fonc.2022.918959 (PMC9337862; doi:10.3389/fonc.2022.918959)
Supplement: Supplementary file 4 [file Table_1.docx]

Supplementary Table 1. Clinical characteristics of 3 patients with MB.

|  | #1 | #2 | #3 |
| --- | --- | --- | --- |
| Age (years) | 4 | 7 | 5 |
| Gender | Female | Male | Male |
| Histopathological types | classic | classic | classic |
| WHO Grade | Ⅳ | Ⅳ | Ⅳ |
| Mass size (cm) | 4×4×5 | 3×3×4 | 5×4×4 |

Supplementary Table 2. The minimum, maximum and median values of TOP2A expression in 20 types of paediatric brain tumors analyzed by UALCAN.

| tumor | minimum | maximum | median |
| --- | --- | --- | --- |
| ATRT | 0.084 | 55.142 | 21.554 |
| CHDM | 0.352 | 13.656 | 9.333 |
| CPP | 0.148 | 3.721 | 0.574 |
| DNT | 0.054 | 2.655 | 0.521 |
| DIPG | 2.442 | 48.906 | 8.808 |
| ES | 16.996 | 52.313 | 30.884 |
| EPMT | 0.02 | 24.851 | 3.622 |
| CRANIO | 0.241 | 5.163 | 1.682 |
| NFIB | 0.132 | 2.392 | 0.718 |
| TT | 1.111 | 21.23 | 4.614 |
| SCHW | 0.409 | 5.122 | 2.367 |
| PNET | 0.455 | 115.156 | 29.109 |
| PLGG | 0.011 | 3.263 | 0.617 |
| PHGG | 0.109 | 68.274 | 14.381 |
| PBL | 13.16 | 78.329 | 20.507 |
| MNG | 0.14 | 12.197 | 3.716 |
| MBL | 1.068 | 98.296 | 36.362 |
| GNOS | 0.024 | 0.615 | 0.04 |
| GNG | 0.027 | 1.88 | 0.332 |
| GMN | 10.33 | 46.629 | 29.811 |
| ATRT, Atypical Teratoid Rhabdoid Tumor; CHDM, Chordoma; CPP, Choroid plexus papilloma; CRANIO, Craniopharyngioma; DIPG, Diffuse intrinsic pontine glioma; DNT, Dysembryoplastic neuroepithelial tumor; EPMT; Ependymoma; ES, Ewings Sarcoma; GMN, Germinoma; GNG, Ganglioglioma; GNOS, Glial-neuronal tumor not otherwise specified (NOS); MBL, Medulloblastoma; MNG, Meningioma; NFIB, Neurofibroma/Plexiform; PBL, Pineoblastoma; PHGG, High-grade glioma/astrocytoma (WHO grade III/IV); PLGG, Low-grade glioma/astrocytoma (WHO grade I/II); PNET, Supratentorial or Spinal Cord primitive neuroectodermal; TT, Teratoma; SCHW, Schwannoma. | | | |
